# Isolation and Identification of Post-Transcriptional Gene Silencing-Related Micro-RNAs by Functionalized Silicon Nanowire Field-effect Transistor

**DOI:** 10.1038/srep17375

**Published:** 2015-11-30

**Authors:** Kuan-I Chen, Chien-Yuan Pan, Keng-Hui Li, Ying-Chih Huang, Chia-Wei Lu, Chuan-Yi Tang, Ya-Wen Su, Ling-Wei Tseng, Kun-Chang Tseng, Chi-Yun Lin, Chii-Dong Chen, Shih-Shun Lin, Yit-Tsong Chen

**Affiliations:** 1 Department of Chemistry, National Taiwan University, Taipei 106, Taiwan; 2 Institute of Atomic and Molecular Sciences, Academia Sinica, P.O. Box 23-166, Taipei 106, Taiwan; 3 Department of Life Science, National Taiwan University, Taipei 106, Taiwan; 4 Institute of Biotechnology, National Taiwan University, Taipei 106, Taiwan; 5 Department of Computer Science, National Tsing Hua University, Hsinchu 300, Taiwan; 6 National Nano Device Laboratories, Hsinchu 300, Taiwan; 7 Institute of Physics, Academia Sinica, Taipei 115, Taiwan; 8 Agricultural Biotechnology Research Center, Academia Sinica, Taipei 115, Taiwan

## Abstract

Many transcribed RNAs are non-coding RNAs, including microRNAs (miRNAs), which bind to complementary sequences on messenger RNAs to regulate the translation efficacy. Therefore, identifying the miRNAs expressed in cells/organisms aids in understanding genetic control in cells/organisms. In this report, we determined the binding of oligonucleotides to a receptor-modified silicon nanowire field-effect transistor (SiNW-FET) by monitoring the changes in conductance of the SiNW-FET. We first modified a SiNW-FET with a DNA probe to directly and selectively detect the complementary miRNA in cell lysates. This SiNW-FET device has 7-fold higher sensitivity than reverse transcription-quantitative polymerase chain reaction in detecting the corresponding miRNA. Next, we anchored viral p19 proteins, which bind the double-strand small RNAs (ds-sRNAs), on the SiNW-FET. By perfusing the device with synthesized ds-sRNAs of different pairing statuses, the dissociation constants revealed that the nucleotides at the 3′-overhangs and pairings at the terminus are important for the interactions. After perfusing the total RNA mixture extracted from *Nicotiana benthamiana* across the device, this device could enrich the ds-sRNAs for sequence analysis. Finally, this bionanoelectronic SiNW-FET, which is able to isolate and identify the interacting protein-RNA, adds an additional tool in genomic technology for the future study of direct biomolecular interactions.

Micro-ribonucleic acids (miRNAs) with lengths of 21 to 22 nucleotides have sequence specificities that guide RNA-induced silencing complexes to cleave the complementary messenger RNAs (mRNAs), resulting in post-transcriptional gene silencing (PTGS)^1^,^2^. This is an important mechanism in controlling the expression of specific genes during the development of an organism^3^. Most miRNA detection methods, such as microarray, Northern blot, and reverse transcription-quantitative polymerase chain reaction (RT-qPCR), are designed for probing single-stranded RNAs^4^,^5^ but are time-consuming and laborious. The viral p19 proteins form dimers and sequester double-strand small RNA duplexes (ds-sRNAs, including miRNA/miRNA*, where miRNA* represents the complementary anti-sense miRNA) in host cells, leading to blocking the host cell RNA interference (RNAi) defense mechanism and prevent the viral RNAs from being digested by gene silencing mechanism^6^. Therefore, p19 is a good candidate for concentrating the ds-sRNAs in an RNA mixture to characterize the miRNAs expressed in a cell or organism. Some reports have adopted p19 as a screening tool to study miRNA expression in cancer cells or tissues; however, these approaches are not reusable and require isotopes to enhance sensitivity. Additionally, they lack real-time detection^7^,^8^.

To characterize the miRNA profile expressed in cells, we applied silicon nanowire field-effect transistor (SiNW-FET) biosensors that provide ultra-sensitive, real-time, and reversible detection^9^,^10^,^11^,^12^. In previous studies, SiNW-FETs were used to detect the interaction between biomolecules^13^ and to sense the virus DNA^14^ or the presence of a specific miRNA from cell extracts^15^ with the femtomolar sensitivity by modifying the SiNWs with complementary oligonucleotides. Therefore, SiNW-FET has the potential in detecting the miRNA expressed in minute levels from an extracted mixture.

To rapidly screen the target-receptor interactions, we applied reusable SiNW-FET devices with reversible surface functionalization based on our previously developed strategy (Supplementary Fig. S1)^16^,^17^,^18^. The conductance of the SiNW-FET is determined by the electric field generated from the molecules surrounding the nanowire; henceforth, the SiNW-FET is very sensitive in monitoring the interaction among biomolecules in a real-time mode. In addition, the anchorage of the receptor molecules on the SiNW-FET surface is reversible; therefore, we could not only analyze the target-receptor interaction but also elute the bound target-receptor complex for analysis.

## Results and Discussion

**Figure 1:**
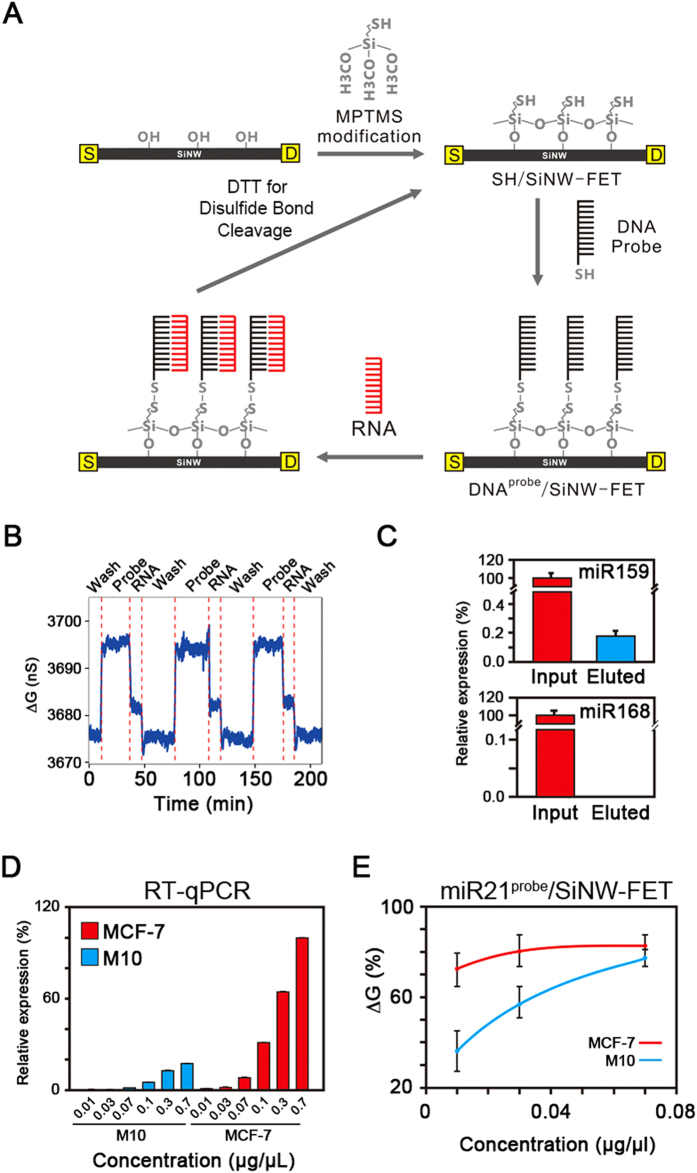
Detection of the endogenous miRNA by SiNW-FET. (**A**) A flow diagram of a reusable DNA^probe^/SiNW-FET device. The MPTMS-modified SiNW-FET (SH/SiNW-FET) provides reversible disulfide bonding sites for the DNA^probe^ tagged with a thiol group at the 3′ end (DNA^probe^/SiNW-FET). After the targeted miRNAs bind to the DNA^probe^/SiNW-FET, the bound DNA^probe^-miRNA complex can be eluted by flushing dithiothreitol (DTT) to break the disulfide bond, returning the device surface to SH/SiNW-FET. (**B**) The electrical conductance changes (ΔGs) of SH/SiNW-FET during repeated cycles of DTT-buffer washing, miR159^probe^ modification, and RNA binding (0.3 μg/μL total RNA extracted from *Arabidopsis*). (**C**) miRNA159 in the Input and Eluted mixtures. After using miR159^probe^/SiNW-FET, the relative amounts of miR159 (upper panel) and miR168 (lower panel) in the bound fraction (Eluted) to the total RNA (Input) extracted from *Arabidopsis* were analyzed by RT-qPCR. (**D,E**) Comparison of the detection limits between RT-qPCR and SiNW-FET. We determined the amounts of miR21 expressed in different concentrations of total RNA extracted from cancer cell lines, MCF-7 and M10, by (**D**) RT-qPCR with specific primers or (**E**) miR21^probe^/SiNW-FET.

The expressions of RNAs are under strict regulation and usually in a small quantity except those house-keeping genes. RT-qPCR is the most sensitive technique to identify the expression of a specific RNA from a mixture owing to its precise amplification procedure. To verify the detection sensitivity and target selectivity of a SiNW-FET in probing the miRNAs of interest from total extracted RNA, we anchored the single-strand DNAs (ss-DNAs) onto a 3-mercaptopropyl-trimethoxysilane (MPTMS)-modified SiNW-FET (referred to as SH/SiNW-FET) via disulfide bonding (referred to as DNA^probe^/SiNW-FET) (Fig. 1A) and perfused the DNA^probe^/SiNW-FET with extracted RNA for the selective binding of the complementary miRNA to the DNA^probe^. We then eluted the bound DNA^probe^-miRNA complexes using dithiothreitol (DTT) to reduce the disulfide bonds and returned the SiNW-FET surface to its original state for device reusability.

To detect the miR159 (5′-UUUGGAUUGAAGGGAGCUCUA-3′), a miRNA which regulates the plant development and fertility^19^, from the total RNA of *Arabidopsis*, we linked a synthesized miR159^probe^ onto the SiNW-FET. In the repeated cycles of electrical measurements (Fig. 1B), the reproducible changes in conductance (ΔGs) of an SH/SiNW-FET in the serial processes of immobilizing miR159^probe^, introducing the total RNA, and eluting the miRNA159/miR159^probe^ complex demonstrated the reusability of the SiNW-FET biosensor. To confirm and quantify the miRNA of interest present in the eluted mixtures and the total extracted RNA, we performed RT-qPCR with specific primers against miR159 (Fig. 1C). Due to the small sensing surface area of the SiNW-FET, the bound miR159 was 0.18% of the miR159 in the total extracted RNA. As a control test, miR168 presents in the total extracted RNA, but not in the eluted complexes. These results demonstrate the specificity of the DNA^probe^/SiNW-FET in recognizing the target miRNA and we could elute the bound oligonucleotides for qPCR amplification or sequencing analysis.

MiRNAs guide the RNA-induced silencing complex to cleave the complementary messenger RNAs, resulting in the PTGS to control the expression level of a specific gene^3^. Henceforth, the information about the relative amount of a specific miRNA in different cells is valuable in understanding the genetic control. To verify that the SiNW-FET is capable of monitoring differential expression of the same miRNA in different cells, we quantified miR21, which is an miRNA that is highly expressed in breast cancer^20^, from the total RNA isolated from an MCF-7 cell line (derived from breast cancer)^21^,^22^ and an M10 cell line (derived from normal mammary epithelial cells). Figure 1D shows that, using RT-qPCR, MCF-7 had an ~5-fold higher miR21 expression level than M10, while the detection limits in both cell lines are ~0.07 μg/μl of the total RNA. In contrast, the miR21^probe^/SiNW-FET was able to detect miR21 from both samples with a total RNA concentration at 0.01 μg/μl (Fig. 1E) and MCF-7 had an ~3-fold higher miR21 level than that of M10. Therefore, both approaches (RT-qPCR and SiNW-FET) demonstrate a similar difference in the expression level of miR21 in these two cell lines. Using RT-qPCR to quantitate the amount of an RNA sample, we need to convert the extracted RNA to cDNA first and then amplify the oligonucleotide segments of interest with specific primers. The detection limit and target sensitivity might be lost during the conversion process but are greatly improved by the amplification procedure. In contrast, a SiNW-FET monitors the direct interaction between the designate miRNA in the total RNA extract and the complementary DNA^probe^ modified on the SiNW-FET; henceforth, a SiNW-FET exhibits high probing sensitivity and detection efficiencies in sample amount and time spent. A recent report even shows that SiNW-FET could have a limit of detection of 1 zeptomole in detecting the miRNA from cancer cell extracrs^23^. These measurements demonstrate that the detection sensitivity of a SiNW-FET biosensor is comparable to RT-qPCR in monitoring differentially expressed genes and support that this SiNW-FET device can be applied to detect the minute expression of a specific miRNA from a mixture.

**Figure 2:**
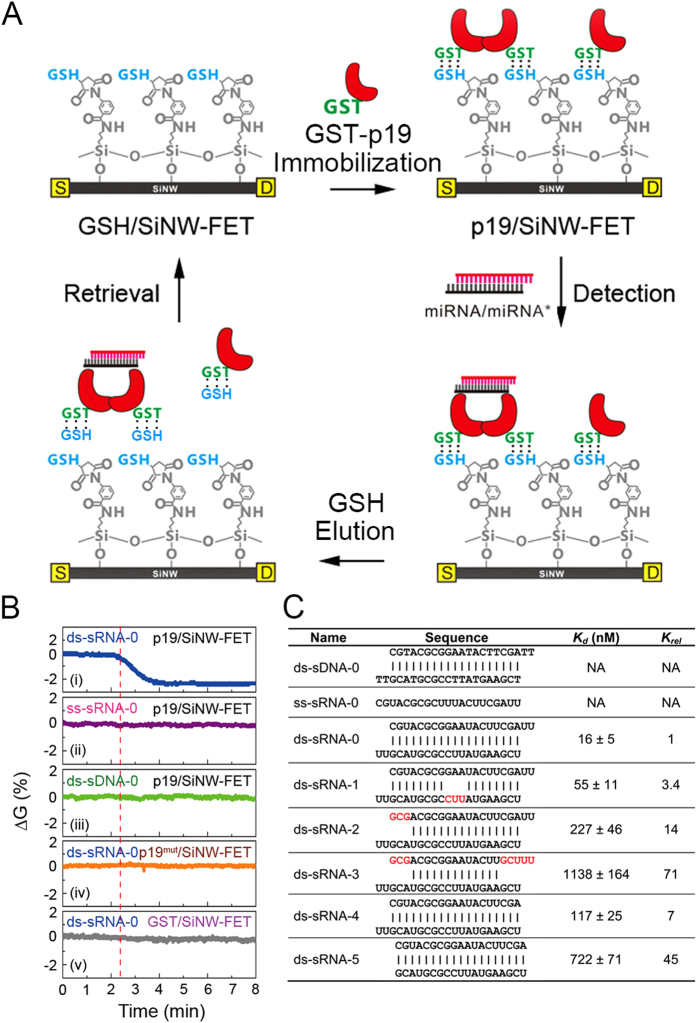
Discriminating the secondary structures of ds-sRNA by p19/SiNW-FET. (**A**) A flow diagram of a reusable p19/SiNW-FET using the GSH/GST association-dissociation. The process includes the immobilization of GST-p19 on a GSH/SiNW-FET to form p19/SiNW-FET, the application of ds-sRNA (miRNA/miRNA*) to bind p19, and the elution of the GST-p19-ds-sRNA complexes with ≥1 mM GSH. (**B**) The responses of p19/SiNW-FET to various forms of nucleic acids. The normalized ∆Gs were measured by introducing 1 μM of a 21-nucleotide solution in (i). perfectly matched ds-sRNA form (ds-sRNA-0, the structure depicted in (**C**)); (ii). ss-sRNA form (ss-sRNA-0); (iii). ds-sDNA form (ds-sDNA-0) to a p19/SiNW-FET; (iv). p19 replaced by a binding-deficient mutant (p19^mut^); and (v). p19 absent in the tests. The vertical red-dotted line indicates the addition of samples. (**C**) The dissociation constant (K_d_) of p19 to various 21-nucleotide nucleic acids with different mismatch pairings. The ΔGs of p19/SiNW-FET to different concentrations of nucleic acids were used to determine the K_d_ values (Supplementary Fig. S5), which were then normalized to that of the ds-sRNA-0 (K_rel_). Nucleotides marked in red indicate the mispaired bases.

To demonstrate the sequestration of the ds-sRNAs by p19, we modified the SiNW-FET with glutathione (referred to as GSH/SiNW-FET) to support the binding of glutathione S-transferase-conjugated p19 (GST-p19) (referred to as p19/SiNW-FET) (Fig. 2A)^17^,^18^,^24^. We performed electric measurements in phosphate solution (containing 240 μM NaH_2_PO_4_ and 760 μM Na_2_HPO_4_ at pH 7.4 with NaOH) with a Debye-Hückel screening length of *λ*_*D*_ = 6.1 nm to sufficiently cover the p19-ds-sRNA complex, which is ~6 nm to the SiNW-FET surface^25^,^26^. The binding of the negatively charged GST (pI ~ 6.72) or GST-p19 (pI ~ 5.9) to the *n*-type GSH/SiNW-FET caused prominent decreases in the normalized ΔG (0.19% and 0.55%, respectively) (Supplementary Fig. S2). It is noteworthy that in the biosensing measurements with SiNW-FETs, the charge redistribution helps the SiNW-FETs to detect target-receptor interactions taking place at the top end of surface linking molecules, which could be much longer than the Debye-Hückel screening length. With charge redistribution, SiNW-FETs are able to detect remote molecular interactions, which would otherwise not be possible^27^. In addition, by using an electrical field perpendicular to the SiNW surface, the structural ordering of surface molecules can be accomplished to improve the sensitivity, reliability, and reproducibility of SiNW-FET biosensors^28^.

As shown in Fig. 2B,C, we tested the binding specificity of p19 to various forms of small nucleic acids by applying 21-nucleotide oligonucleotides in different pairing states to the p19/SiNW-FET and measuring the corresponding ΔGs. The 21-nucleotide segment in the form of a perfectly paired ds-sRNA with two nucleotides at the 5′-overhang (denoted by ds-sRNA-0 with the structure being depicted in Fig. 2C) reduced ΔG by ~2% (Fig. 2B(i)). The decrease of ΔG is due to the gating effect of the negatively charged phosphate backbone of ds-sRNA-0 to the *n*-type p19/SiNW-FET. In sharp contrast, the same sequence in ss-sRNA (ss-sRNA-0, Fig. 2B(ii)) or double-stranded DNA (ds-sDNA-0, Fig. 2B(iii)) forms did not affect the ΔG. To further confirm the specificity of p19 in the interaction with ds-sRNA, we constructed a binding-deficient mutant, p19^mut^, which had four conserved amino acids, P_37_, L_39_, H_40_, and W_42_ and were mutated to S, G, S, and G, respectively (Supplementary Fig. S3)^26^,^29^. The lack of observable ΔG (Fig. 2B(iv)) indicated negligible binding between ds-sRNA and p19^mut^; in addition, the p19^mut^ could not bind the fluorescence-tagged ds-sRNA as p19 did (Supplementary Fig. S4). These results reveal that p19 selectively binds ds-sRNA and the 2′ hydroxyl group of RNA plays a significant role in the association with p19.

To characterize whether p19/SiNW-FET could differentiate the mismatched pairing in ds-sRNA, we synthesized five ds-sRNAs (ds-sRNA-1~5 with their structures being listed in Fig. 2C) with mismatched nucleotides or blunt ends and calculated the dissociation constants (K_d_) between the p19/SiNW-FET and ds-sRNA-x (x = 0–5). By plotting the measured ΔGs as functions of the sample concentration and fitting these data to the Langmuir adsorption isotherm model (Fig. S5)^30^,^31^, the K_d_ of the p19-ds-sRNA-0 complex was 16 ± 5 nM, similar to the previously reported values^32^,^33^,^34^, and the detection limit was 1 nM with signal-to-noise (S/N) ≥3. For ds-sRNA-1 with three mismatched bases at nucleotide positions of 9–11, the K_d_ was slightly increased to 55 ± 11 nM; for ds-sRNA-2 and ds-sRNA-3, of which both have mismatched bases at the termini, the K_d_ values were greatly increased to 227 ± 46 nM and 1138 ± 164 nM, respectively. For ds-sRNA-4 with a single blunt end and ds-sRNA-5 with two blunt ends, the K_d_ values were increased largely to 117 ± 25 and 722 ± 71 nM, respectively. These results support previous reports that the two tryptophans of a p19 monomer interact with the overhangs at the 3′-ends of miRNA/miRNA* duplexes^29^. In addition, the termini of the RNA duplex are more critical in binding to p19 than the central region, and our p19/SiNW-FET biosensor exhibits binding affinities that can distinguish various ds-sRNA secondary structures.

**Figure 3:**
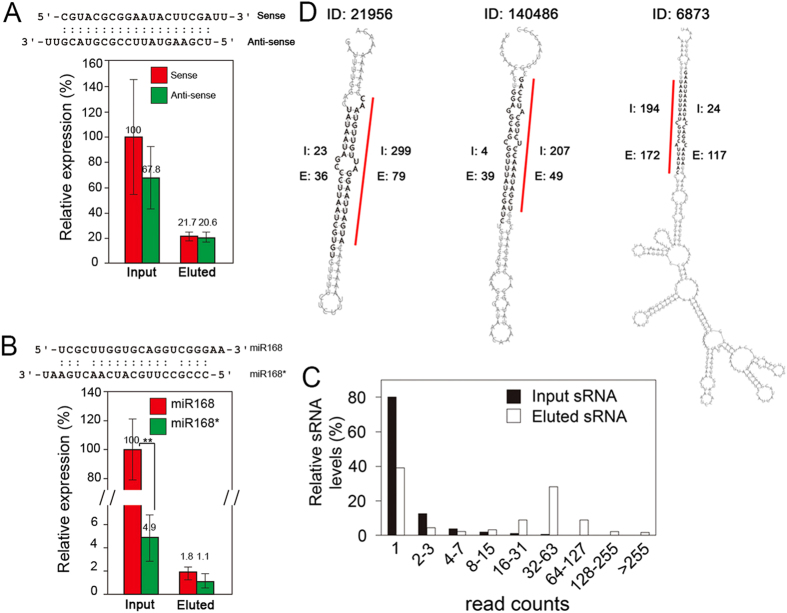
p19/SiNW-FET sequestration enriches the ds-sRNAs. We perfused the p19/SiNW-FET with sRNA (Total) from synthesized ds-sRNA-0 (**A**) or extracted RNA from *Arabidopsis* (**B**) or *Nicotiana benthamiana* (**C**); we then eluted the bound sRNA (Eluted) from p19/SiNW-FET and analyzed the relative amounts of the designated sRNA by (**A,B**) RT-qPCR or (**C**) a deep sequencer. (**A**) The relative amounts of sense and anti-sense strands of ds-sRNA-0 in the total and eluted fractions. (**B**) The relative amounts of miR168 (sense) and miR168* (anti-sense) in the extracted and bound fractions. (**C**) Proportions of the analyzed sRNAs with different read counts. The number of each sRNA sequenced was counted (read counts) and binned with a power of 2; the number of sRNAs in each binning group was normalized to the total number of sRNA counted. (**D**) Predicted structures of three pre-miRNAs yielding the corresponding paired ds-sRNAs captured on p19/SiNW-FET. According to the genome of *N. benthamiana*, nine genes (Table S1) might transcribe pre-miRNAs which have 2^nd^ structures yielding paired ds-sRNAs (bases in bold) identified from the Elute fraction. The red lines indicate the matured miRNA forms. The digits beside each sRNA segment (bases in bold) are the counts of the corresponding sRNA segment appearing in the Input (I) or Eluted (E) sRNA sample. The data (mean ± standard deviations) were the averages of three independent experiments and ** indicates the *p*-value < 0.01. A magnified image of Fig. 3D is shown in Supplementary Fig. S7 for easier reading.

To verify that p19/SiNW-FET could selectively sequester ds-sRNAs from an RNA mixture, we perfused the p19/NW-FET with synthesized ds-sRNA-0 (Fig. 3A) or extracted RNA from *Arabidopsis* (Fig. 3B) and analyzed the relative amount of sRNA (the sense vs. anti-sense strands of ds-sRNA-0) by RT-qPCR. Perfusion of the synthesized ds-sRNA-0 or total extracted RNA across the p19/NW-FET induced a decrease in the conductivity (Supplementary Fig. S6). Figure 3A shows that the normalized amounts of the sense and anti-sense strands of ds-sRNA-0 in the input mixture were 100 and 67.8%, respectively; whereas the relative amounts in the eluted sample were 21.7 and 20.6%, respectively. These results revealed that the sense and anti-sense strands of ds-sRNA-0 are not all paired in the input mixture; however, after p19 sequestration, the amounts of the sense and anti-sense strands are almost of equal amounts suggesting that the sRNA are mostly paired. Unlike the synthesized ds-sRNA-0, endogenous ds-sRNAs degraded quickly; so the total RNA (input) contains very few miR168* (4.9%) relative to the miR168 (regarded as 100%) as shown in Fig. 3B; however, the eluted sRNAs contains 1.8 and 1.1% of miR168 and miR168*, respectively. The nearly equal amounts of sense and anti-sense miRNAs in the eluted samples demonstrate the binding specificity of p19 to ds-sRNA.

To verify p19/SiNW-FET is useful in enriching the miRNA/miRNA* duplexes specifically in the total RNA extracted from *N. benthamiana*, we dissociated the GSH-GST association to release the p19-ds-sRNA complexes from the SiNW-FET surface and used a next generation sequencing (NGS) technique to analyze the sequence of each ss-sRNA in the total (Input) and captured (Eluted) samples (Fig. 3C). The abundance of a specific ss-sRNA is determined by the times of the identified sequences (read counts). In total extracted RNA, 80% of the 4,653,979 ss-sRNAs identified was sequenced one time (1 read count), and only 1.1% had counts of ≥16. For the eluted ss-sRNAs (total count is 199,001), 39% was sequenced one time and >50% had counts of ≥16. The decrease and increase in the fractions of low and high sequence counts, respectively, support the ability of p19 in selectively enriching certain sRNAs. To demonstrate that the eluted sRNA contains ds-sRNA, we paired the possible ds-sRNA from the sequences obtained according to the following algorithms: (1) read counts of >30, (2) two nucleotides at the 3′ overhang on both termini, (3) less than four mismatched pairing, and (4) permissible G:U pairing in the RNA structure. The above requirements (1)–(3) were decided according to the results of Fig. 2C which shows the different association constants between p19 and ds-sRNAs with different pairing statuses; the requirement (4) is a general rule for RNA pairing. Using these criteria, we identified 459 and 3,752 possible ds-sRNA pairs present in the input and eluted sRNA samples, respectively. Considering the total number of ss-sRNA sequenced in the input (4,653,979) and eluted (199,001) fractions, the ds-sRNA fraction dramatically increased after p19 enrichment (i.e., 459/4,653,979 in the input and 3,752/199,001 in the eluted). These results confirm that most of the enriched sRNAs after p19/SiNW-FET sequestration are ds-sRNAs that were derived from the sRNAs with low abundances in the cells.

Single RNA strands, like transfer RNAs or ribosomal RNAs, form special hairpin structures for various functions. To generate the miRNAs for gene silencing, each transcribed pre-miRNA forms a hairpin structure with the sense and anti-sense strands of the prospective miRNA pair, though not necessary in a perfect match status. The pre-miRNA is then digested to produce miRNA/miRNA* pairs and the miRNA will then bind to the complementary mRNA to activate the PTGS for gene silencing^35^. To recognize the possible pre-miRNA genes responsible for the ds-sRNAs that were identified in the eluted mixture, we used the RNAfold server (Vienna RNA WebServers; http://rna.tbi.univie.ac.at) to predict the possible structures of several novel *N. benthamiana* pre-miRNA genes. Because the genomic database of *N. benthamiana* has not yet been completed, we could identify only nine potential pre-miRNA genes, which could generate the corresponding paired ds-sRNAs identified from the sequence results (Table S1). The paired sRNA species must be in the same pre-miRNA contig and at the stem of the hairpin structure. Figure 3D shows three predicted RNA secondary structures of the nine potential pre-miRNA genes. The counts of these three miRNA/miRNA* pairs in the total extracted RNA were 299/23, 207/4, and 194/24, respectively, indicating that after digestion of the pre-miRNAs in cells, most miRNA* were degraded. However, after p19 sequestration, the miRNA/miRNA* ratio tends to approaching unity, i.e. 79/36, 49/39, and 172/117, respectively, for the three predicted structures. These results further support that the p19/SiNW-FET binds ds-sRNAs with high affinity and specificity.

In conclusion, the reversible design of a SiNW-FET device facilitates the isolation and identification of ds-sRNAs from samples with low biologically relevant concentrations, demonstrating that a SiNW-FET can be used as a biosensor for the fast analysis of sRNAs in genomic studies. In addition, the direct interaction between a nucleic acid probe, immobilized on the SiNW-FET, and the target nucleic acid provides a biosensing tool with a sensitivity at the femtomolar level, which is suitable for most biomedical diagnoses^36^. Although we have improved the reusability of a SiNW-FET device in detecting biomolecular interactions via a reversible surface modification method for calibratable and quantitative analyses^17^, we could not ignore the technical challenges and environmental confrontations for SiNW-FETs, e.g., neutral molecule detection, sensing in a high salt buffer, and high-throughput multiplexed sensing^11^.

In this report, we have shown that the p19/SiNW-FET can not only offer highly sensitive detection for miRNA but also probe the low abundance of intermediate forms of miRNA/miRNA* duplexes with high affinity and specificity. Therefore, with appropriate modifications to SiNW-FET, our system has great potential for biomedical applications in detecting protein-RNA interactions with high sensitivity and reusability. Finally, this bionanoelectronic device, which is able to isolate and identify the interacting protein-RNA, adds an additional tool in genomic technology for the future study of direct biomolecular interactions.

## Methods

### Fabrication of SiNW-FET devices

The SiNW-FETs were fabricated from silicon-on-insulator (SOI) wafers^17^,^27^,^28^,^37^, composed of a layer of high-quality single-crystal silicon (device layer), a layer of buried oxide (BOX layer), and the bulk substrate. These SOI wafers are currently state-of-the-art in the production of metal-oxide-semiconductor field-effect-transistors (MOSFETs). In this study, 4-inch *n*-type SOI wafers containing a 50-nm-thick device layer and a 400-nm-thick BOX layer with a device layer resistivity of approximately 100 Ω·cm and a phosphor doping concentration of 4 × 10^19^ cm^−3^ were used. After standard electron-beam lithographic and photolithographic fabrication procedures, the SOI chip was thermally oxidized to form a SiO_2_ insulating layer (~10 nm in thickness) on top of the SiNW surface to prevent the charge transfer between analyte molecules and the SiNW-FET. The advantages of this SiO_2_ dielectric layer are not only providing an insulator layer to prevent electrical leakage to the aqueous buffer solution during biosensing measurements, but also facilitating the coupling of a PDMS channel onto the SiNW-FET chip for subsequent experiments without contaminating the SiNW-FET devices. Each chip (14 × 14 mm^2^ in dimension) contains eight SiNW-FET devices (L 6 μm × W 200 nm × T 50 nm for each SiNW, Supplementary Fig. S1). The contact pads (Ni/Au = 10/50 nm) were fabricated by photolithography and were attached directly to the Si device layer.

### Reversible surface modification of the SOI chip

The reversible surface modification of SiNW-FETs can be realized via either the formation of disulfide bonds^16^, or the association-dissociation of GSH/GST^17^,^18^ on the SOI surface.

In the immobilization of DNA^probe^ on the SiNW-FET surface (as shown in Fig. 1A), 1% 3-mercaptopropyltrimethoxysilane (MPTMS) in ethanol was first pumped into a polydimethylsiloxane (PDMS) microfluidic channel (L 6.25 × W 0.5 × H 0.05 mm^3^), which was designed to couple the SiNW-FET device arrays, with a syringe pump at a flow rate of 300 μL/hr for 30 min. After MPTMS was modified on the SiNW-FET (denoted by SH/SiNW-FET), ethanol was guided into the PDMS microfluidic channel to wash the FET devices for 10 min. It is noted that ethanol solution might swell the PDMS channel and MPTMS could also be modified on the PDMS channel walls; to minimize this disadvantage, the swollen PDMS channel could be replaced with a new one for the following experiments. Subsequently, 500 mM dithiothreitol (DTT) in 1× PBS (containing 138 mM NaCl, 2.7 mM KCl, 8 mM Na_2_HPO_4_, 1.5 mM KH_2_PO_4_, pH 7.4 with NaOH) was pumped into the PDMS channel for 30 min to reduce any possible disulfide bonding between the thiol groups of MPTMS. The SH/SiNW-FET was then flushed with 1× PBS for 20 min. Next, a 1× PBS solution containing 1 μM DNA (disulfate DNA-dimer) and 100 mM DTT was pumped into the PDMS microfluidic channel. Finally, the sulfhydryl group of the DNA^probe^ reacted with the thiol group of MPTMS to anchor the DNA^probe^ on the MPTMS/SiNW-FET via the formation of a disulfide bond (referred to as DNA^probe^/SiNW-FET). After each electrical measurement, where miRNAs bound specifically to the DNA^probe^/SiNW-FET, the DNA-miRNA complexes were removed by cleaving the disulfide bond with DTT (500 mM in 1× PBS) for 2 hr to return the device surface to SH/SiNW-FET, thus making the SiNW-FETs reusable.

In the modification of GSH on the SiNW-FET surface (Fig. 2A), the SOI chip containing eight SiNW-FET devices was immersed in 1% 3-aminopropyltrimethoxysilane (APTMS) in ethanol for 30 min and then heated at 110 °C for 10 min to form a self-assembled layer of APTMS on the SOI surface. A linker of 3-maleimidobenzoic acid N-hydroxysuccinimide ester (MBS), prepared in a 1:9 mixture of dimethyl sulfoxide and 1× PBS, was applied to react APTMS through the formation of an amide bond. Subsequently, the SOI chip was immersed in 1× PBS containing 1 mM GSH for 30 min to induce bonding between the sulfhydryl group of GSH and the maleimide group of MBS (to form a GSH/SiNW-FET), washed with 1× PBS, and blow-dried with N_2_.

### Electrical measurement

In the electrical measurements with a SiNW-FET, the sample molecules were diluted in 0.1× PS (phosphate solution, containing 240 μM NaH_2_PO_4_, 760 μM Na_2_HPO_4_, pH 7.4 with NaOH) with the Debye-Hückel screening length of *λ*_*D*_ = 6.1 nm. The sample solution, pumped into a PDMS microfluidic channel with a syringe pump at a flow rate of 0.3 mL/h, was delivered to the SiNW-FET surface. All sensing measurements were conducted using a detection system that combined a current preamplifier (1211, DL Instruments) and a lock-in amplifier (SR830, Stanford Research Systems) operating at a source-drain voltage (V_ds_) of 30 mV, a modulation frequency of 79 Hz, and a time constant of 100 ms^16^,^17^,^18^,^27^,^28^. An Ag/AgCl electrode (MF2052, BASi) connected to the PDMS microfluidic channel could be used as a solution gate. To minimize noise, the Ag/AgCl electrode was maintained at ground potential throughout the electrical measurements.

The charge sensitivity of a SiNW-FET is in principle the same as that of thin-film FETs, provided that the thickness of the thin film does not exceed the Debye length of the semiconducting channel. In that case, the operation of both SiNW-FETs and thin-film FETs are in the fully depleted regime.

### Amplification and quantification of the sRNA sequence using RT-qPCR

The RT-qPCR method used to amplify and quantify the gene expression levels has been described previously^5^. For the reverse transcription (RT) reaction, a combination of 4.5 μL of the p19/SiNW-FET eluents, 50 nM of a stem-loop RT primer, 0.25 mM of dNTP, 20 units reverse transcriptase, 1× reverse transcriptase buffer, 10 mM of DTT, and 4 units of an RNase inhibitor was prepared. The reactions were incubated at 16 °C for 30 min, followed by pulsed RT for 60 cycles at 30 °C for 30 s, 42 °C for 30 s, and 50 °C for 1 s to produce the first-strand cDNA. The RT reaction was terminated by heating at 85 °C for 5 min.

qPCR was performed using the Universal Probe Library assay (Roche Diagnostics, Indianapolis, Indiana, USA) to amplify and quantify the cDNA synthesized from the miRNA captured by the p19/SiNW-FET. For the PCRs, 2.7 μL of the cDNA mixture was combined with 0.5 μM of each forward and reverse primer, 0.1 μM of Universal Probe Library Probe no. 21, and 1× Master Mix solution. The PCR amplification curves were generated using an initial denaturation at 95 °C for 10 min, followed by 45 cycles of 95 °C for 5 s, 60 °C for 30 s, and 72 °C for 1 s. The data were collected and analyzed using a LightCycler 480 real-time PCR instrument (Roche Molecular Systems, Pleasanton, CA, USA).

For the PCR reaction, the universal reverse primer was 5′-GTGCAGGGTCCGAGGT-3′; the forward and RT primers for miR159 were 5′-GGCTCATTTGGATTGAAGGGA-3′ and 5′-GTTGGCTCTGGTGCAGGGTCCGAGGTATTCGCACCAGAGCCAACTAGAGC-3′; for miR168 were 5′-TGGCCCGCCTTGCATCAA-3′ and 5′- GTTGGCTCTGGTGCAGGGTCCGAGGTATTCGCACCAGAGCCAACATTCAG-3′; for miR168* were 5′-TGGCCCGCCTTGCATCAA-3′ and 5′-GTTGGCTCTGGTGCAGGGTCCGAGGTATTCGCACCAGAGCCAACATTCAG-3′; for ds-sRNA-0 sense strand were 5′-GGCTCACGTACGCGGAATACT-3′ and 5′- GTTGGCTCTGGTGCAGGGTCCGAGGTATTCGCACCAGAGCCAACAATCGA-3′; for ds-sRNA-0 anti-sense strand were 5′-GGCTCATCGAAGTATTCCGCG-3′ and 5′- GTTGGCTCTGGTGCAGGGTCCGAGGTATTCGCACCAGAGCCAACAACGTA-3′.

### Dissociation constant

The dissociation constant (K_d_) of the p19-ds-sRNA complex was determined by a least-squares fit of the 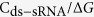 vs. 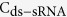 data (Supplementary Fig. S5C) to the Langmuir adsorption isotherm model of^30^,^31^


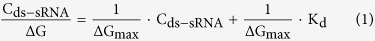


where 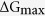 is the saturated 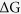 at C_ds–sRNA_ ≥ 200 nM (Supplementary Fig. S5B). As plotted in Supplementary Fig. S5C, the fit to the experimental data yielded K_d_ = 15.9 ± 4.8 nM of the p19-ds-sRNA complex.

### Molecular biology

We amplified the full-length p19 from infectious clones of the CymRSV^32^,^38^ by PCR using the p19-F1 (5′-CCCCGGATCCATGGAACGAGCTATACAAGG-3′) and p19-R1 (5′-CGCCCCTCGAGCTACTCGCTTTCTTCTTTGAAGGC-3′) primers and inserted into the pGEX-4T-1 bacterial plasmid (GE Healthcare, Waukesha, WI, USA) for protein expression. To construct the mutated p19 (p19^mut^), we used p19-Mut-FW (5′-GACGAAAGTTCGAGTGGCTCTGAGGGGAGGCTACATCAC-5′) and p19-Mut-Rev (5′-GTGATGTAGCCTCCCCTCAGAGCCACTCGAACTTTCGTC-3′) primers for PCR-mediated mutagenesis and inserted the product into pGEX-4T-1. To express the proteins, we transformed the BL21 (DE3) strain of *Escherichia coli* with these constructs and induced the protein expression by adding isopropyl ß-D-1-thiogalactopyranoside (IPTG, 1 mM) into the lysogeny broth (LB) medium.

We extracted the total RNA from the leaves of 2-week-old *Arabidopsis thaliana* (col-0) seedlings using the Trizol reagent (Invitrogen, Carlsbad, CA, USA). The 21-nucleotides positive-stranded sRNA (5′-CGUACGCGGAAUACUUCGAUU-3′) and negative-stranded sRNA (5′-UUGCAUGCGCCUUAUGAAGCU-3′) were synthesized by MDBio (Taipei, Taiwan). The 5′-ends of the sRNAs were phosphorylated and a Cy5 fluorescent label was added to the 5′-end of the positive-stranded sRNA. The related 21-nucleotide DNA primers, including the positive-stranded DNA oligo (5′-CGTACGCGGAATACTTCGATT-3′) and negative-stranded DNA oligo (5′-TTGCATGCGCCTTATGAAGCT-3′), were synthesized and used as controls for the p19 binding assay.

### sRNA analysis and miRNA prediction

We sequenced the sRNAs either isolated from *N. benthamiana* or eluted from the p19/SiNW-FET by a next generation sequencer (NGS), Illumina HiSeq 2000 system (Illumina, San Diego, CA, USA). We first removed the sequence errors in the reads by filtering through the *N. benthamiana* genome version 0.44 contigs (Niben.genome.v0.4.4.contig.fasta)^39^; the clean reads were used to determine the read counts (weight) and the identity of the sRNA species.

## Additional Information

**Accession codes:** The raw sRNA reads reported in this paper are available in the NCBI Short Read Archive under accession numbers SRR1555764 (input sample), and SRR1555765 (eluted sample).

**How to cite this article**: Chen, K.-I. *et al.* Isolation and Identification of Post-Transcriptional Gene Silencing-Related Micro-RNAs by Functionalized Silicon Nanowire Field-effect Transistor. *Sci. Rep.* **5**, 17375; doi: 10.1038/srep17375 (2015).

## Supplementary Material

Supplementary Information
