# Supplementary material for: Isolation and Identification of Post-Transcriptional Gene Silencing-Related Micro-RNAs by Functionalized Silicon Nanowire Field-effect Transistor
Source: Sci Rep. 2015 Nov 30;5:17375. doi: 10.1038/srep17375 (PMC4663627; doi:10.1038/srep17375)
Supplement: Supplementary Information [file srep17375-s1.pdf]

Supporting Information

Isolation and Identification of Post-Transcriptional Gene Silencing-Related Micro-RNAs by Functionalized Silicon Nanowire Field-effect Transistor

Kuan-I Chen[1,2], Chien-Yuan Pan[3], Keng-Hui Li[1], Ying-Chih Huang[4], Chia-Wei Lu[4], Chuan-Yi Tang[5], Ya-Wen Su[6], Ling-Wei Tseng[1], Kun-Chang Tseng[1,2], Chi-Yun Lin[1], Chii-Dong Chen[7], Shih-Shun Lin[4,8]*, Yit-Tsong Chen[1,2]*

[1]Department of Chemistry, National Taiwan University, Taipei 106, Taiwan

[2]Institute of Atomic and Molecular Sciences, Academia Sinica, P.O. Box 23-166, Taipei 106, Taiwan

[3]Department of Life Science, National Taiwan University, Taipei 106, Taiwan

[4]Institute of Biotechnology, National Taiwan University, Taipei 106, Taiwan

[5]Department of Computer Science, National Tsing Hua University, Hsinchu 300, Taiwan

[6]National Nano Device Laboratories, Hsinchu 300, Taiwan

[7]Institute of Physics, Academia Sinica, Taipei 115, Taiwan

[8]Agriculture Biotechnology Research Center, Academia Sinica, Taipei 115, Taiwan

**Corresponding author: E-mail: ytcchem@ntu.edu.tw; Fax: +886-2-3366-8671; E-mail: linss01@ntu.edu.tw; Fax: +886-2-3366-6001*

Table S1. Predicted pre-miRNAs yielding the paired sRNAs

No.	miRNA ID	Location[a] (nucleotide)	Reads[b]				*N. benthamiana* genomic contig ID
			Total		Eluted		
			5'-strand	3'-strand	5'-strand	3'-strand	
1	21956	16326-16385	23	299	36	79	Niben044Scf00022111
2	140486	3698-3783	4	207	39	49	Niben044ctg26199327
3	32913	9345-9438	401	628	62	108	Niben044scf00033254
4	21409	9750-9883	428	50231	35	16420	Niben044scf0021673
5	6873	64319-64586	194	24	172	117	Niben044scf00006983
6	37443	31216-31573	6570	924	3703	235	Niben044scf0037814
7	16712	52681-53224	71	0	54	30	Niben044scf00016874
8	11818	238382-239250	2	3	36	44	Niben044scf00011819
9	20111	3622-4500	0	189	33	91	Niben044scf00020348

[a]The position of the predicted miRNA gene bases on the sequence of the genomic contigs of *Nicotiana benthamiana*.

[b]The number of each sRNAs sequenced in the total and eluted sRNA.

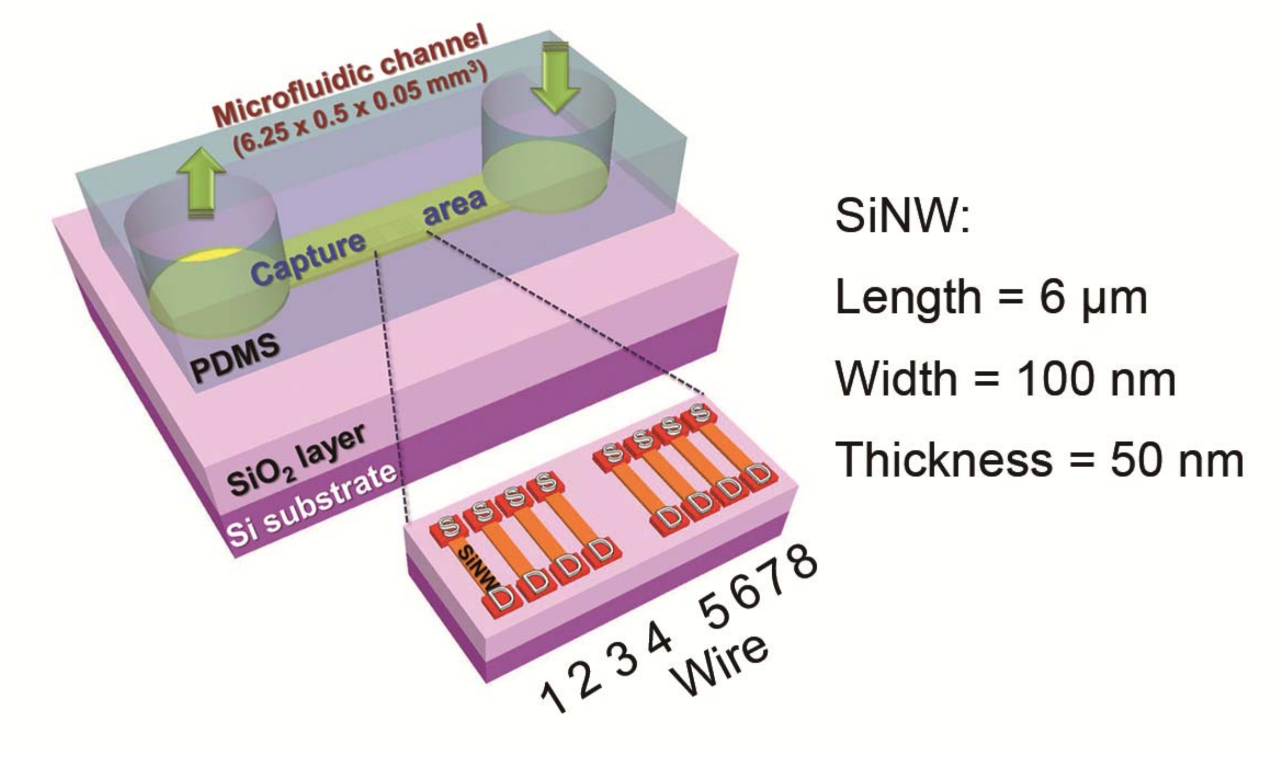

Figure S1. An illustrative representation of eight SiNW-FET devices and the capture area on an SOI chip.

The eight SiNW-FETs are labelled in numerical order. The capture area beneath the PDMS microfluidic channel covers not only minute SiNW-FET surfaces but also the vast surrounding substrate surface. Because a SiO_2 layer was coated on the capture area, the immobilization of receptors (e.g., DNA^{probe} and p19 in this study), the binding of targets (miRNA and ds-sRNA), and the elution of the receptor-target complexes (DNA-miRNA and p19-ds-sRNA) occurred on the entire region of the capture area.

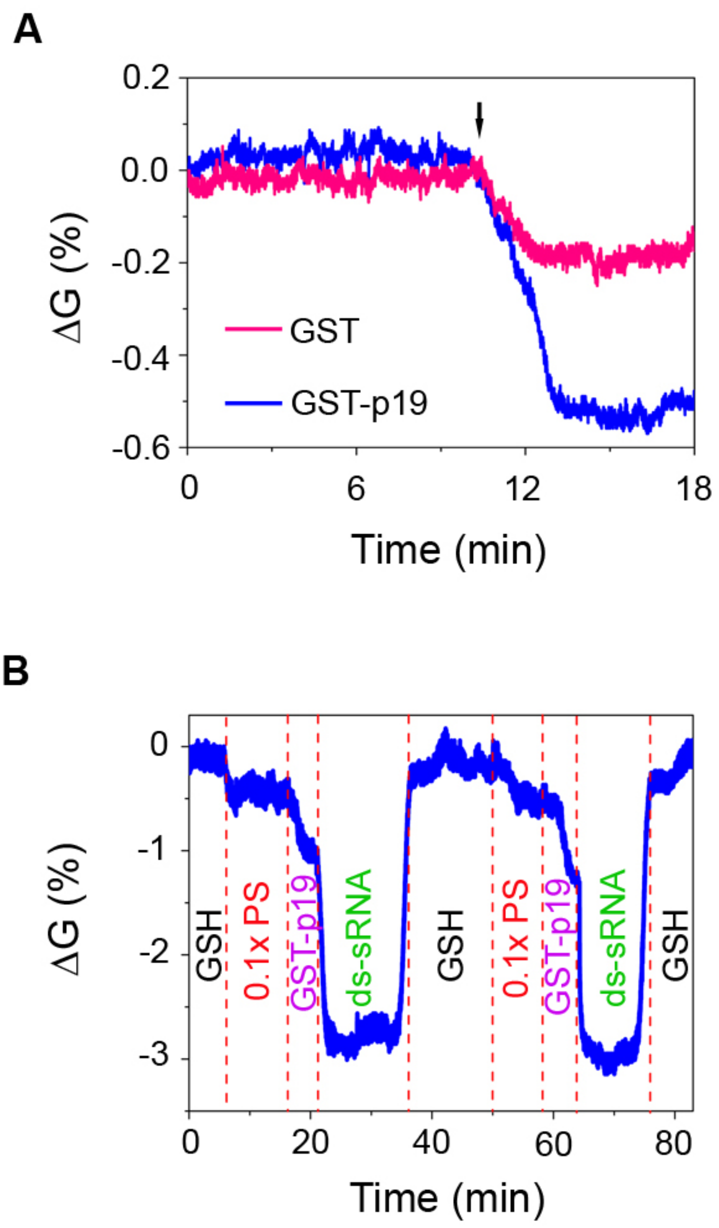

Figure S2. The binding tests of ds-sRNAs to a p19/SiNW-FET.

(A). The electrical conductance changes (ΔGs) of a GSH/SiNW-FET responded to the associations of GST (red trace) and GST-p19 (blue trace), respectively. (B). A reusable SiNW-FET biosensor was demonstrated by the reproducibility of the electrical conductance changes (ΔGs) of a GSH/SiNW-FET endured in the two complete cycles of GSH washing, 0.1× PS flushing, GST-p19 immobilization, and ds-sRNA binding. The reproducible ΔG at each repeated step demonstrated the operating stability and device reusability of the SiNW-FET system.

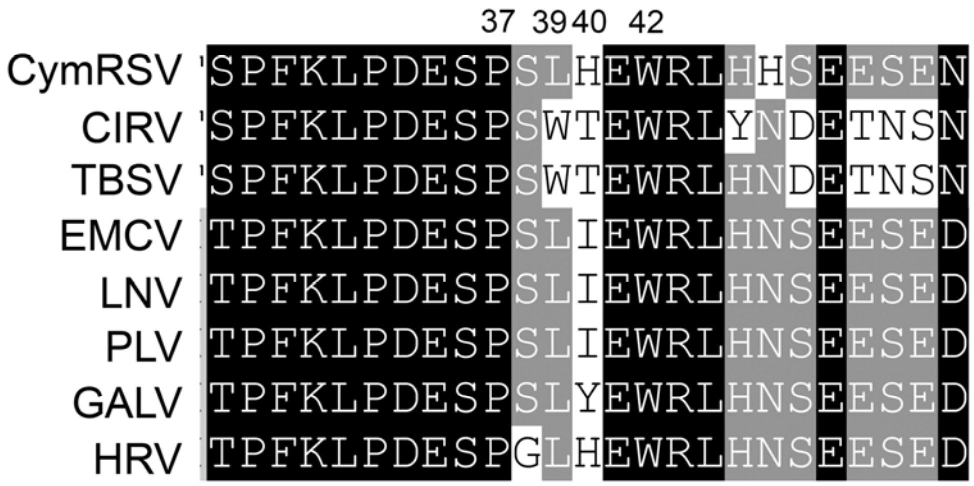

Figure S3. The p19 sequence alignment.

The partial sequence alignment of p19 proteins from *Cymbidium ringspot virus* (CymRSV), *Tomato bushy stunt virus* (TBSV), *Eggplant mottled crinkle virus* (EMCV), *Lisianthus necrosis virus* (LNV), *Havel river virus* (HRV), *Grapevine Algerian latent virus* (GALV), and *Pear latent virus* (PLV). The black boxes represent the highly conserved amino acids, and the gray boxes indicate the conserved amino acids. The numbers on top of panel indicate the positions of the amino acids that were substituted in this study.

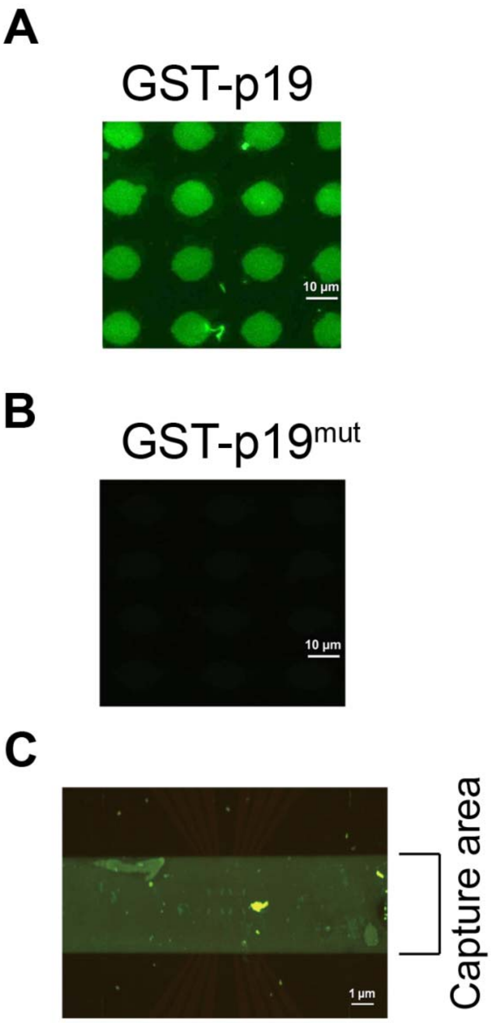

Figure S4. Successful surface modification proved by fluorescence imaging examination.

(A). Binding of 21-nucleotide ds-sRNA to the p19-modified micropatterns. Green fluorescence indicates the successful association of 5'-fluorescein isothiocyanate (FITC)-ds-sRNAs with the p19-modified micropatterns. (B). No fluorescence could be observed for the 21-nucleotide ds-sRNA binding to the $p19^{mut}$-modified micropatterns, suggesting that the four mutated amino acids of $p19^{mut}$ are crucial to the binding of the ds-sRNA. (C). Visualization of the ds-sRNA captured by p19 over the entire capture area (as illustrated in Figure S1). Green fluorescence indicates that 5'-FITC-ds-sRNAs were captured by the p19 immobilized on the surface of the entire capture area.

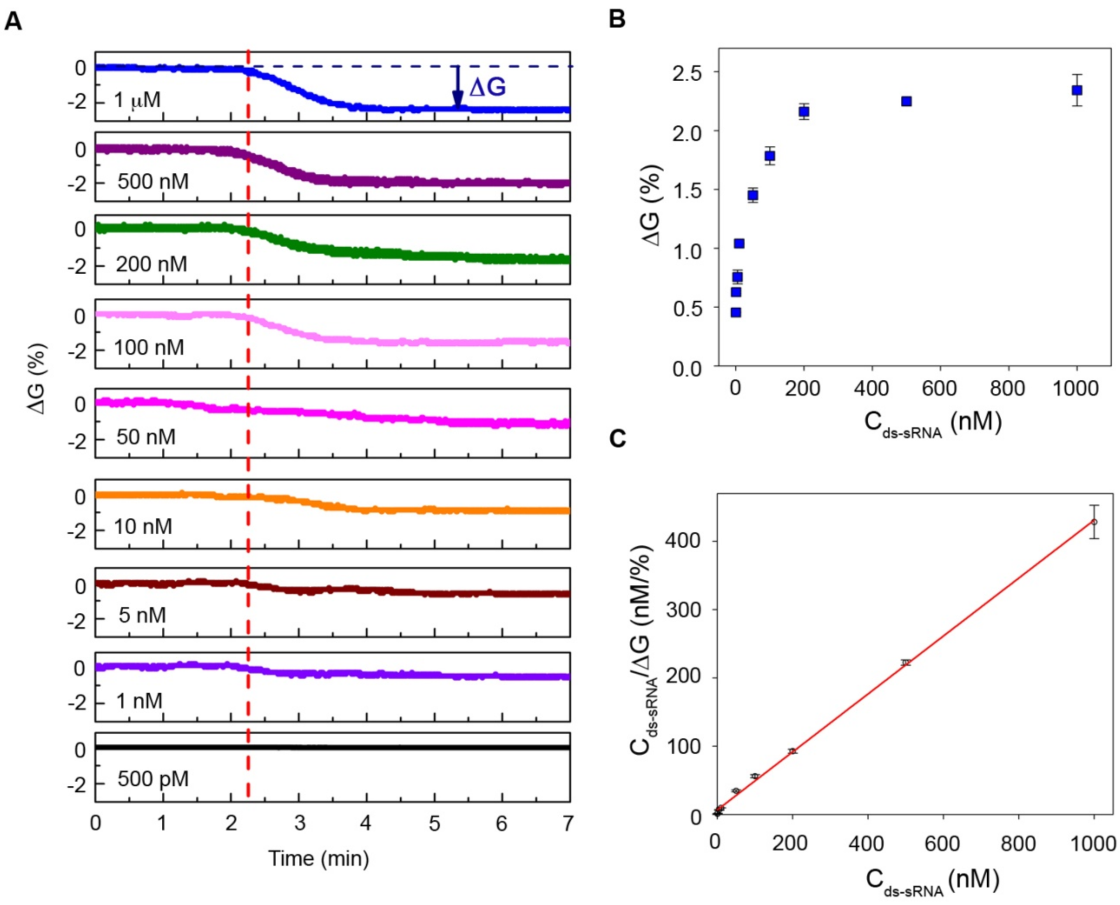

Figure S5. Determination of the dissociation constant (K_d) of the p19-ds-sRNA complex.

(A). The electrical conductance changes (ΔGs) of a p19/SiNW-FET after introducing various concentrations of ds-sRNAs at 500 pM–1 μM. The vertical red-dotted line indicates the addition of ds-sRNA samples. (B). The ΔG values are presented as a function of ds-sRNA concentration ($C_{ds-sRNA}$) where the data points are taken from (A). (C). A least-squares fit of the $C_{ds-sRNA} / \Delta G$ vs. $C_{ds-sRNA}$. The data points fit to the Langmuir adsorption isotherm model (Eq. 1) yielded $K_d = 15.9 \pm 4.8$ nM for the p19-ds-sRNA complex.

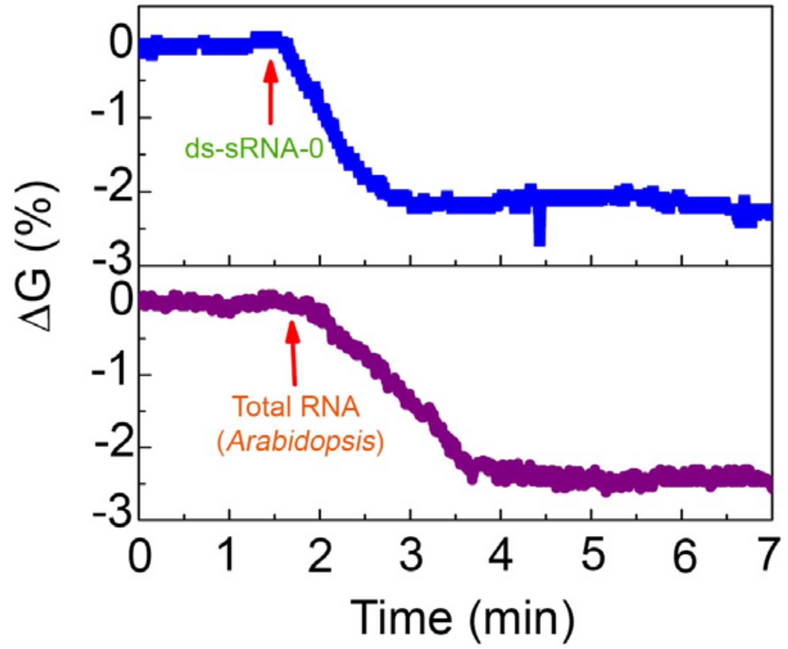

Figure S6. Detections of synthetic ds-sRNAs and total extracted RNA by p19/SiNW-FET. The normalized ΔG of a p19/SiNW-FET in the detection of (upper panel) synthetic ds-sRNA-0 or (lower panel) the total RNA extracted from *Arabidopsis thaliana*. The decrease of ΔG is due to the gating effect of the negatively charged phosphate backbone of ds-sRNA-0 to the *n*-type p19/SiNW-FET.

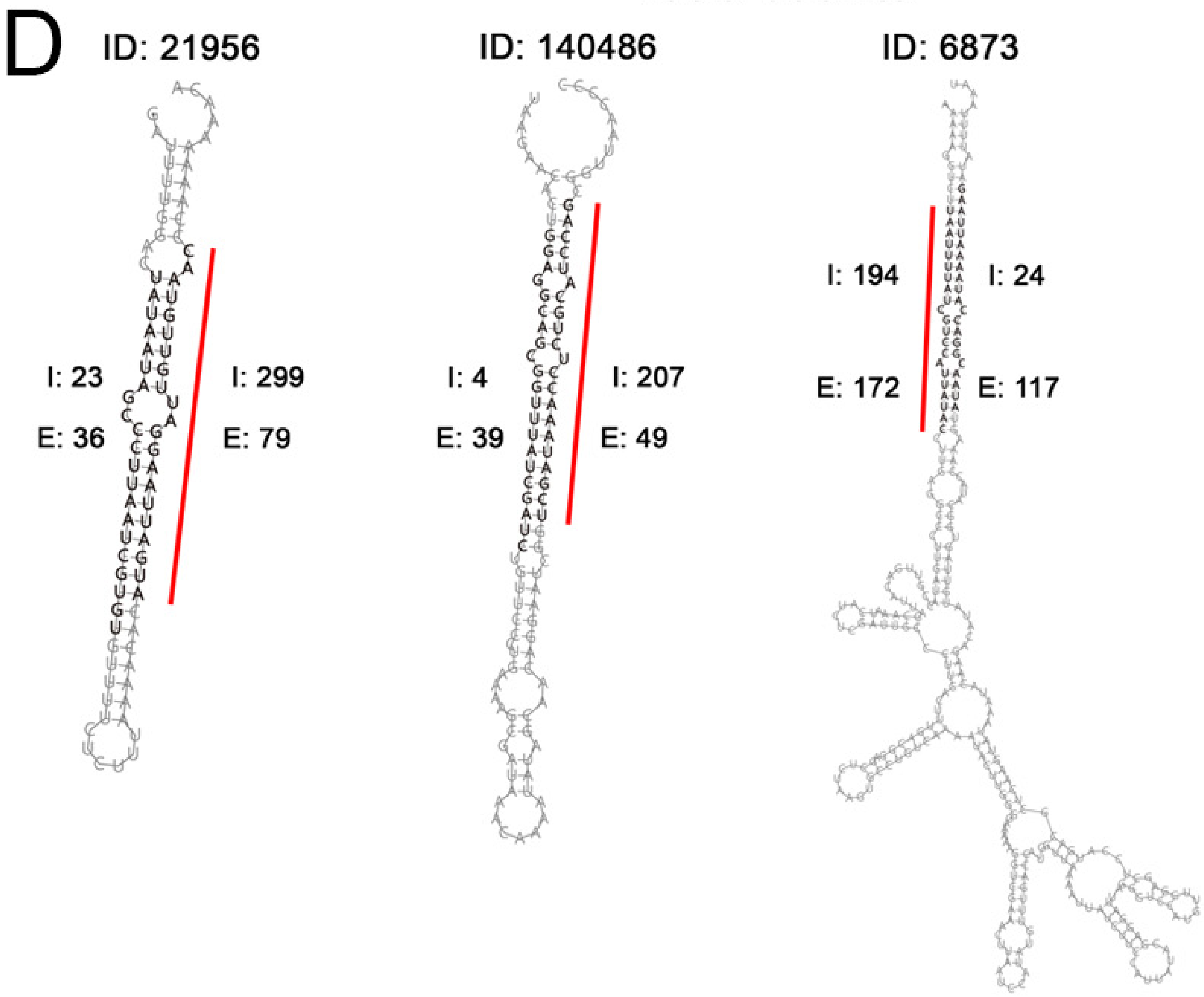

Figure S7. A magnified image of Figure 3D for easier reading.
